# In vitro CT visualization of the biopsy location by filling the core biopsy needle with contrast media

**DOI:** 10.1177/02841851211041831

**Published:** 2021-10-22

**Authors:** Per Thunswärd, David Eksell, Håkan Ahlström, Anders Magnusson

**Affiliations:** Department of Surgical Sciences – Radiology, 8097 Uppsala University, Uppsala, Sweden

**Keywords:** CT, Biopsy, Contrast Agents-Other, Experimental Investigations

## Abstract

**Background:**

When performing computed tomography (CT)-guided biopsy procedures with non-disposable, automatic biopsy instruments, the actual course of the biopsy needle is not registered.

**Purpose:**

To evaluate the ability to visualize the sampling location after CT-guided biopsy in vitro using a novel method, where the space between the inner needle and the outer cannula in a core biopsy needle is filled with contrast media; and to compare the grade of visibility for two different concentrations of contrast media.

**Material and Methods:**

Core needle biopsies were performed in a tissue phantom using biopsy needles primed with two different iodine contrast media concentrations (140 mg I/mL and 400 mg I/mL). Commercially available needle-filling contraptions with sealing membranes were used to fill the needles. Each biopsy was imaged with CT, and the visibility was evaluated twice by three senior radiologists in a randomized order.

**Results:**

The presence of traces was confirmed after biopsy, almost without exception for both concentrations. The visibility was sufficient to determine the biopsy location in all observations with the 400 mg I/mL filling, and in 7/10 observations with the 140 mg I/mL filling. The grade of visibility of the trace and the proportion of the biopsy needle course outlined were higher with the 400 mg I/mL filling.

**Conclusion:**

With CT-guided biopsy in vitro, the sampling location can be visualized using a novel method of priming the biopsy needle with iodine contrast media, specifically highly concentrated contrast media.

## Introduction

Guidance with computed tomography (CT) is frequently used when performing biopsies of lesions in lungs, bones, or deep structures (1). The needle biopsy procedure usually includes the utilization of a coaxial needle that is inserted close to or into the lesion to be sampled, followed by multiple sampling with a cutting core biopsy instrument (2). A commonly used type of biopsy needle consists of a cutting outer cannula surrounding an inner stylus, with a side notch that acts as a sampling chamber. The biopsy needle, in turn, is attached to a spring-loaded gun, permitting tissue sampling in either a semi- or fully automated manner (3).

Semi-automatic biopsy guns (SBGs; often disposable and lightweight), offer the opportunity to perform imaging and thereby verify the needle position (hence, the location of sampling). Automatic biopsy guns (ABGs; especially non-disposable) provide larger tissue sample volumes and a higher rate of success in accuracy and negative predictive values compared to SBGs, but do not offer imaging of the exact location of sampling (3-5). The latter since scanning after firing the biopsy gun (before needle retraction) would imply pronounced metal artifacts and add unjustified radiation dose to the operator's hand.

Since the biopsy procedure is not without risk or inconvenience for patients and is also resource intensive, reducing the proportion of non-specific and non-diagnostic biopsies would be valuable (2). To potentially achieve this, we propose, to the best of our knowledge, a new method of visualizing the sampling locations of non-disposable ABGs. The rationale for using this method, in extension, is to possibly allow for instant identification of non-hits, thus enabling coaxial needle repositioning and the opportunity to take additional biopsies in the same session.

The method is based on the principle that it is possible to fill the tiny space between the inner and outer needle (including the notch) with a contrast agent, which previously has allowed for improved sonographic visibility of core biopsy needles in vitro (6). The main aim of the present study was to evaluate the ability to visualize the sampling locations after CT-guided biopsy in vitro using a novel method, where the space between the inner needle and the outer cannula in a core biopsy needle is filled with contrast media. A secondary purpose was to compare the grade of visibility for two different concentrations of contrast media.

## Material and Methods

### Tissue phantoms, biopsy gun, and needle-filling contraptions

Hemicylinder-shaped and same-sized blood puddings (charcuterie made from, among other things, porcine blood and lard) were used as tissue phantoms. The phantoms were placed one at a time in a plastic basket, with the rounded side facing upwards and strapped to the bottom of the basket. In each phantom, points of insertion were evenly marked out in a standardized manner. Core needle biopsies (CBNs) were performed at those points of insertion through an introducer needle (17 G × 14.6 cm, Co-Axial Introducer Needle; Argon Medical Devices, Inc., Plano, TX, USA). A non-disposable ABG (Pro-Mag™ Ultra, Automatic Biopsy Instrument, Argon Medical Devices, Inc., Plano, TX, USA) with an associated biopsy needle (18G × 20 cm, Pro-Mag™ Biopsy Needle; Argon Medical Devices, Inc., Plano, TX, USA) was used. The biopsy gun was fixed on a metal tripod during the entire biopsy procedure.

**Fig. 1.**
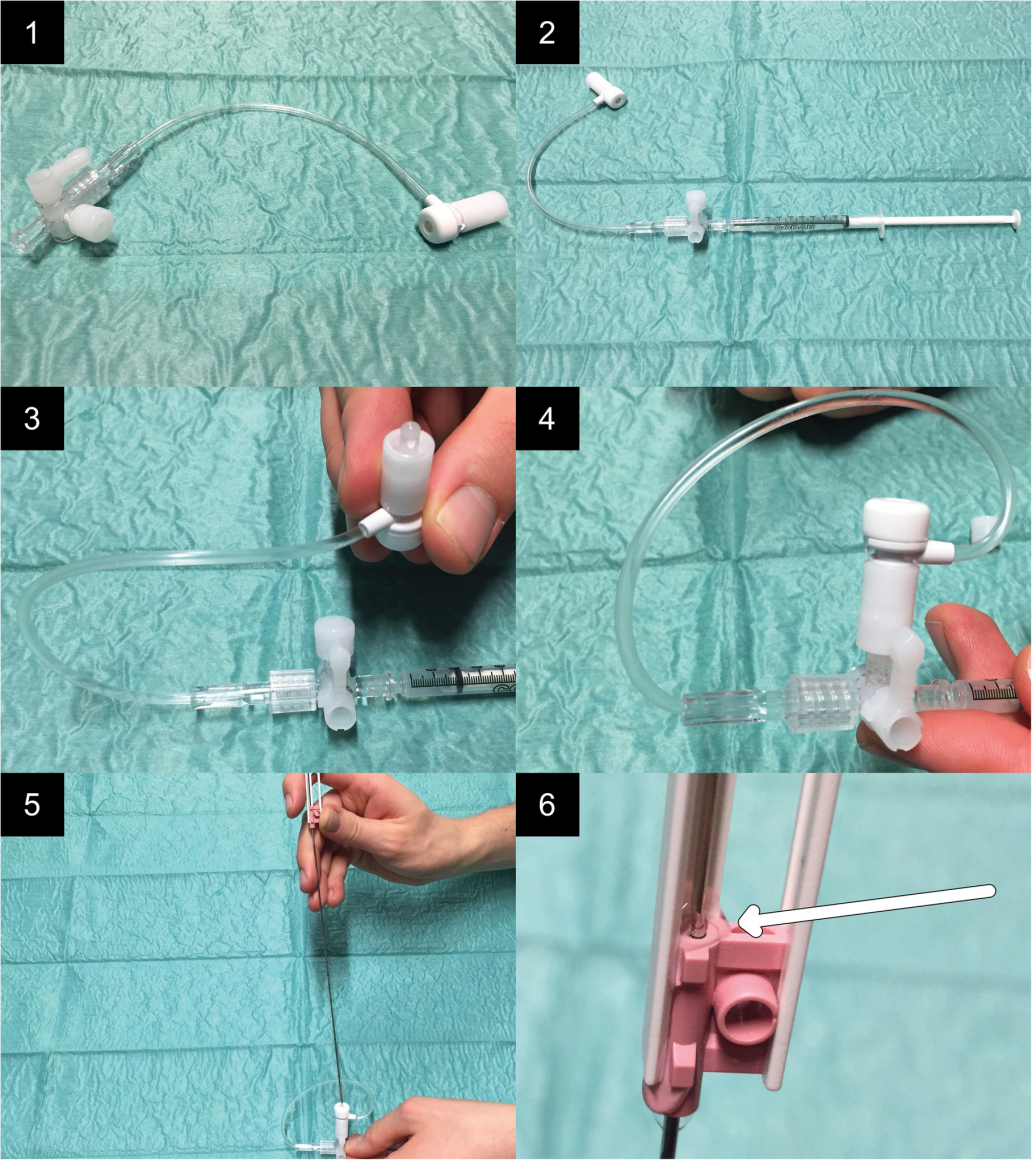
Presentation of the procedure of filling the space between the inner needle and the outer cannula process. 1: The needle-filling contraption before assembling. 2: A syringe (1 mL; Luer slip) is connected to the device. 3: The contraption is filled with the contrast media. 4: The membrane part of the contraption is connected to the three-way tap. 5: The tip of the biopsy needle is pushed through the membrane. 6: As a result of adding pressure with the syringe, contrast media is forced into the space between the inner needle and the outer cannula. Filling of the entire space is confirmed by the outflow of contrast media at the upper orifice (arrow).

Needle-filling contraptions (Check-Flo® Haemostasis Assembly; Cook Medical, Bloomington, IN, USA) were used to fill the space between the inner needle and the outer cannula of the biopsy needles with iodine contrast media (described in Fig. 1). Each contraption consists of a three-way tap connected to a membrane via a plastic tube and is usually used within peripheral intervention. Ten biopsy needles were filled with iohexol (Omnipaque™; GE Healthcare, Stockholm, Sweden), having an iodine concentration of 140 mg I/mL (low concentration). Another 10 were filled with iomeprol (Iomeron®; Bracco S.p.A., Milano, Italy), having an iodine concentration of 400 mg I/mL (high concentration). These two concentrations were chosen because they are the lowest and the highest commercially available. Each contraption, including the hose, was pre-filled with 0.9 mL of the respective contrast media. It was determined that a minimum volume of 0.08 mL was required to fill up the space between the inner needle and the outer cannula of the biopsy needle. For that reason, at least that volume of the contrast media was forced into each biopsy needle via the filling contraption.

### Imaging

Imaging was performed using a multidetector computed tomography scanner (SOMATOM Definition Edge; Siemens Medical Solutions, Forchheim, Germany) by an experienced radiographer specializing in CT. The following settings were used: automatic tube current modulation (CARE Dose 4D); quality reference of 100 mAs; care kV with a reference of 120 kV (fixed); slider at position 7; rotation time = 0.5 s; collimation = 128 × 0.6 mm; pitch = 0.6 with a 50% overlap; kernel = B31 abdomen; gantry tilt angle = 20°.

### Biopsy procedure

**Fig. 2.**
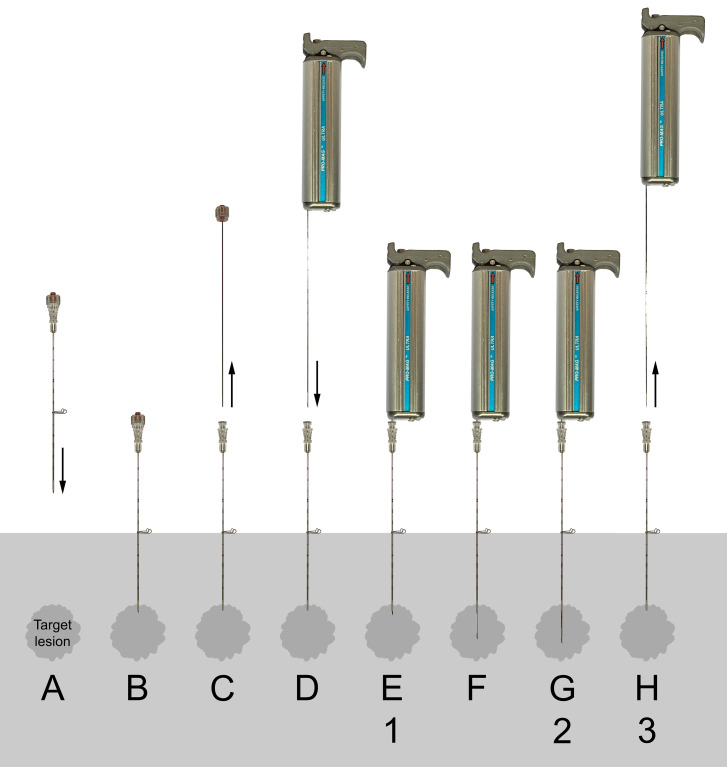
Schematic presentation of the stages (A–H) of the biopsy procedure using an ABG and coaxial technique (numbers 1–3 denote stages imaged with CT and subsequently evaluated by the three assessors): (A, B) the coaxial needle is positioned with the tip just adjacent to, or in the periphery of, the target lesion. (C) The stylet of the coaxial needle is retracted. (D, E) The biopsy needle of the loaded biopsy gun is inserted into the coaxial needle. (F, G) Both the inner stylet and cutting outer cannula are automatically advanced with spring force in a sequential manner when the biopsy gun is fired. (H) The biopsy instrument and needle are finally retracted, and the specimen in the notch harvested. SBGs differ in stages (F) and (G), where the inner stylet is manually inserted to its desired position (F), followed by firing off the biopsy gun, resulting in the advancement of only the outer cannula with spring force (G). ABG, automatic biopsy gun; CT, computed tomography; SBG, semi-automatic biopsy gun.

A total of 20 biopsies were performed, using new biopsy needles for each. In every second biopsy, the biopsy needle was prepared with the low concentration and in every other with the high concentration to reduce the risk of bias due to any possibly occurring inhomogeneities within and between the tissue phantoms. The introducer needle was inserted vertically with the tip at a depth of 1.5 cm. Each biopsy performed included eight different stages (denoted A–H), of which three (denoted 1–3) were imaged (Fig. 2).

### Image processing and evaluation

The image material was subjected to image processing before being presented to the observers. Digital alignment was performed between the stages to allow for complete visualization of the biopsy channel in the same slice. A multiplanar reconstruction (MPR) slab (3 cm) with 3-mm slices for each stage was prepared in Vue PACS version 12.1.6.0071 (Carestream, Rochester, NY, USA).

**Fig. 3.**
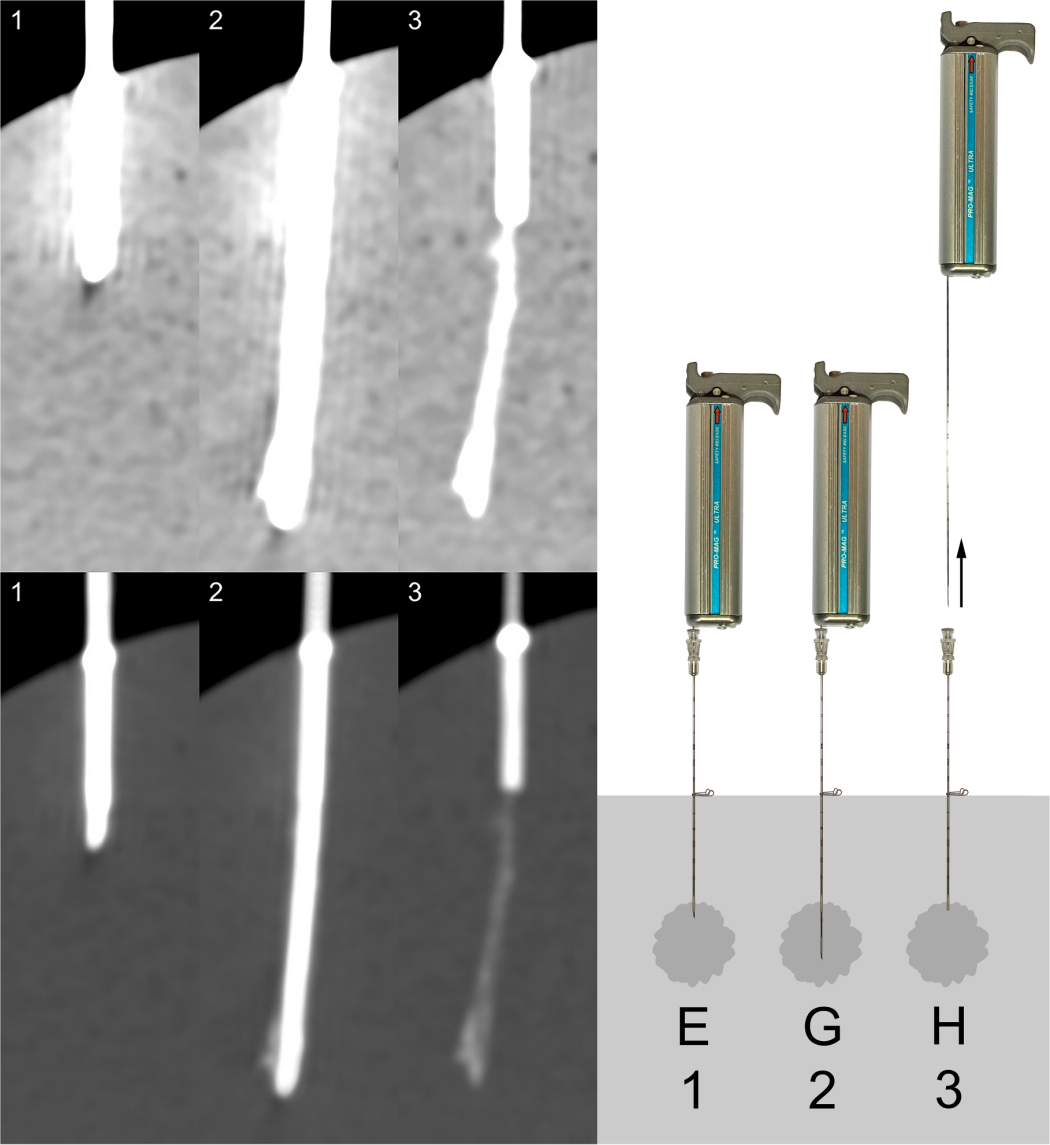
Left: All three stages for one of the punctures (denoted H5 in Fig. 4) in the high concentration series (400 mg I/mL) as presented to the observers (top row: abdominal window; bottom row: bone window). Right: Illustration of depicted stages 1–3 (excerpt from Fig. 2).

Three senior radiologists individually assessed each of the 20 biopsies, blinded and in random order. For each biopsy, all three imaged stages were presented in both the abdominal window (window width = 350 HU, window center = 50 HU) and the bone window (window width = 2000 HU, window center = 500 HU), as exemplified in Fig. 3. All biopsies were assessed twice, resulting in a total of 40 observations per radiologist. For each observation, the radiologists were asked to assess the visibility of any occurring trace and to answer the questions in Table 1. When question 1 was answered with a “No,” variables 2 and 3 were assigned value 0 and variable 4 value “No.”

**Fig. 4.**
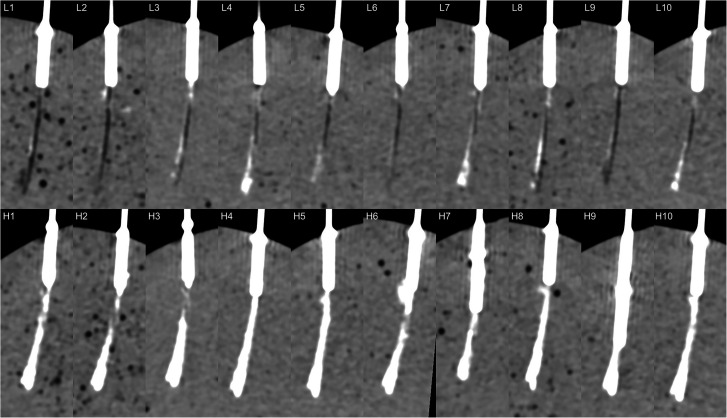
All punctures in stage 3 (as defined in Fig. 2). Top row (L1–10): low concentration (140 mg I/mL); bottom row (H1–H10): high concentration (400 mg I/mL).

**Table 1.** Assessment questions answered by the three senior radiologists for each observation.

Variable	Question	Values
1. Trace visible	Can you distinguish any high attenuating trace along the biopsy needle course?	Yes/No
*If the answer is “Yes” in question 1:*		
2. Grade of visibility	How visible is the high attenuating trace?	VAS: 0–100 mm (not visible – very visible)
3. Proportion outlined	How high proportion of biopsy needle course is outlined by the high attenuating trace?	VAS: 0–100 mm (nothing – the entire course)
4. Quality sufficient	Is the quality of the high attenuating trace altogether sufficient to decide where the biopsy sample was taken?	Yes/No

VAS, visual analogue scale.

### Analysis and statistics

For variables 1 and 4, the frequency and proportion of positive answers (“Yes”) were calculated for each of the assessors and weighted together by using the majority. The two different concentrations were compared using Fisher's exact test.

For variables 2 and 3, the mean of the first and second assessment for each assessor and biopsy performed was calculated. In addition, a weighted together value was calculated for each biopsy performed, in terms of the mean of the first and second assessment for all three assessors. From those mean values, the first, second (median), and third quartiles were calculated; furthermore, unpaired two-sample Wilcoxon tests were performed to compare the two different concentrations. The intra-rater and inter-rater reliability were estimated by calculating the intraclass correlation coefficients (ICC type 3; consistency, two-way mixed, single score).

All statistical tests were two-tailed and performed at 0.05. Analyses were performed using RStudio version 1.4.1106 (RStudio, Inc., Boston, MA, USA).

## Results

**Fig. 5.**
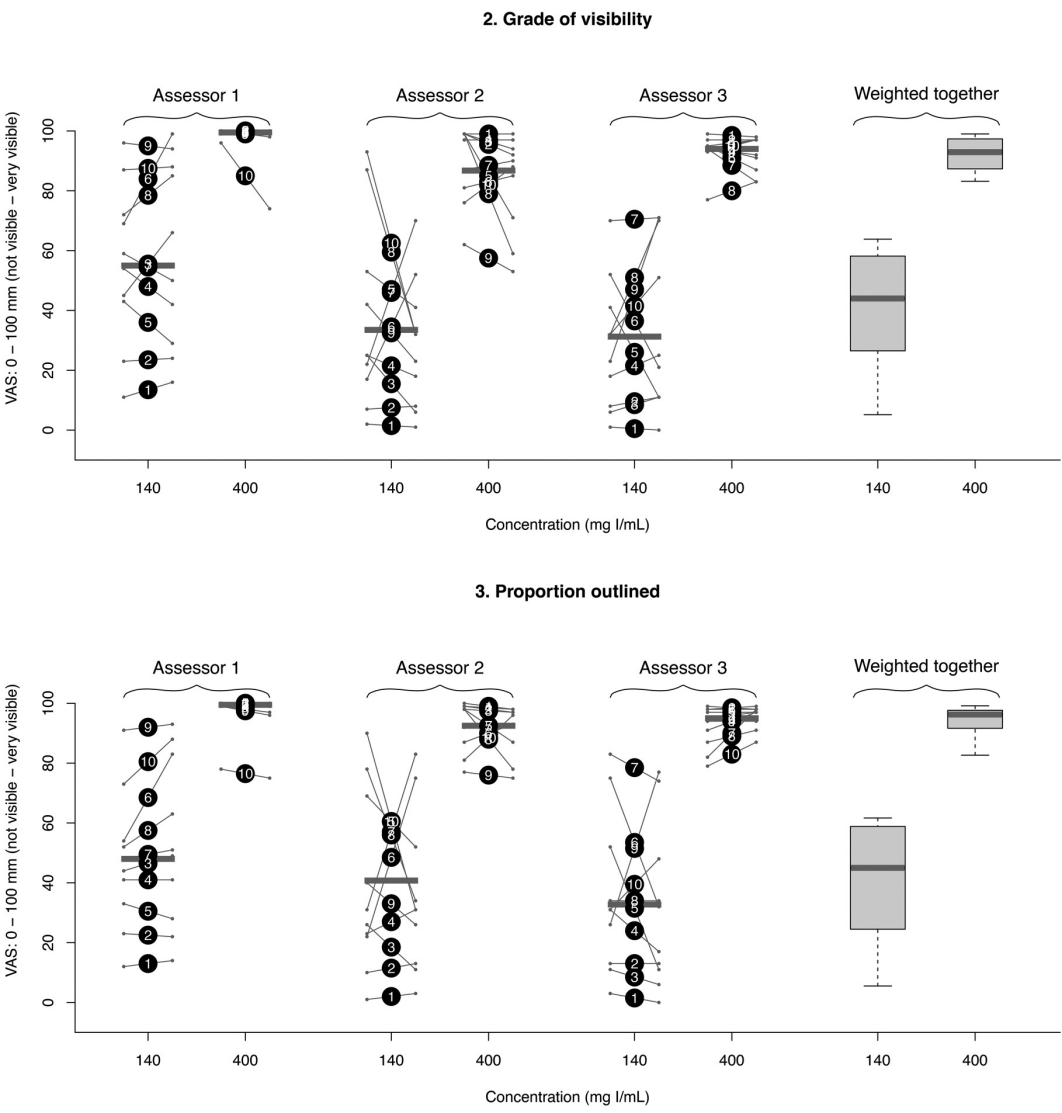
Diagram of the outcome of variables 2 (grade of visibility) and 3 (proportion outlined) for the three assessors separately as dots with interconnecting lines and weighted together as box plots (each grouped by the two different contrast agent concentrations). Big dots represent the mean of the first and second assessments (the numbers 1–10 within correspond to the punctures depicted in Fig. 4). Small dots with interconnecting lines represent the first (to the left) and second (to the right) assessments. Horizontal gray bars represent median values. The lower and upper limits of the boxes represent first and third quartiles, while the lower and upper limits of the whiskers represent the minimum and maximum values.

For the low concentration (140 mg I/mL), a trace was considered as existing (variable 1) in 59 of the 60 assessments and with enough quality to determine where the biopsy sample was taken (variable 4) in 50 of the 60 assessments. For the high concentration (400 mg I/mL), a trace was considered as existing (variable 1) and with sufficient quality to determine where the biopsy sample was taken (variable 4) in all of the 60 assessments. Using high concentration contrast media resulted in a higher grade of visibility and proportion outlined (variables 2 and 3: *P* < 0.001). There were no differences between the two concentrations in the proportion of traces visible (variable 1: *P* > 0.99), or the sufficient quality to determine where the biopsy sample was taken (variable 4: *P*>=0.21). For variables 2 and 3, the intra-observer reliability varied from 0.22–0.99 and the inter-observer reliability from 0.15–0.44. The results are presented in detail in Table 2 (all 4 variables) and in Fig. 5 (variables 2 and 3).

**Table 2.** Results of assessments.*

	Variable	Conc.	Measures	Assessor 1	Assessor 2	Assessor 3	Weighted together
1	Trace visible	140	A1–A2	10/10–10/10	10/10–10/10	10/10–9/10	10/10
		400	A1–A2	10/10–10/10	10/10–10/10	10/10–10/10	10/10
		Comp. 140 vs. 400		*P* > 0.99	*P* > 0.99	*P* > 0.99	*P* > 0.99
2	Grade of visibility	140	M (Q_1_–Q_2_) ICC	55 (14–83) 0.88	34 (17–47) 0.22	31 (13–46) 0.51	44 (27–58) 0.68
		400	M (Q_1_–Q_2_) ICC	100 (100–100) 0.29	87 (82–96) 0.43	94 (92–96) 0.60	93 (88–97) 0.15
		Comp. 140 vs. 400		*P* < 0.001	*P* < 0.001	*P* < 0.001	*P* < 0.001
3	Proportion outlined	140	M (Q_1_–Q_2_) ICC	48 (33–66) 0.93	41 (21–57) 0.20	33 (16–49) 0.51	45 (26–58) 0.61
		400	M (Q_1_–Q_2_) ICC	100 (98–100) 0.99	93 (89–98) 0.40	95 (91–98) 0.58	96 (92–98) 0.44
		Comp. 140 vs. 400		*P* < 0.001	*P* < 0.001	*P* < 0.001	*P* < 0.001
4	Quality sufficient	140	A1–A2	10/10–10/10	7/10–7/10	7/10–9/10	7/10
		400	A1–A2	10/10–10/10	10/10–10/10	10/10–10/10	10/10
		Comp. 140 vs. 400		*P* > 0.99	*P* = 0.21	*P* = 0.21	*P* = 0.21

* Questions 1 and 4: proportion answered “Yes”. Questions 2 and 3: mean VAS value (0–100) of A1 and A2. Variables 1–4 are defined in Table 1.

A1, Assessment 1; A2, Assessment 2; Comp., comparison between the two concentrations (Fisher's exact test for variables 1 and 4; unpaired two-sample Wilcoxon tests for variables 2 and 3); Conc., concentration in mg I/mL; ICC, intraclass correlation coefficients (intra-rater reliability for “Assessor 1–3” columns, inter-rater reliability for “Weighted together” column); M, median; Q_1_–Q_2_, 1st–3rd quartile.

## Discussion

There were two main results of this study. First, filling CBNs with contrast media, almost without exception, rendered high attenuating traces after CT-guided biopsy. Second, the quality of the traces created with a high concentration of contrast media throughout was sufficient to determine where all the biopsy samples were taken from. There was also a higher grade of visibility and proportion of the biopsy site outlined compared with a low concentration of contrast media. To the best of our knowledge, this method has not been described previously.

The ICC values for variables 2 and 3 ranged from poor to excellent for the intra-rater reliability and poor throughout for the inter-rater reliability based on criteria suggested by Koo et al (7). ICC values tend to be low when the variability between subjects observed is low (8). Hence, the relatively low ICC values for the majority of the high concentration assessments could be explained by them all being at the high end of the scale and does not necessarily reflect a lack of intra- and inter-observer agreement. For the dichotomous variables 1 and 4, calculation of the kappa coefficient would have been the natural choice of method to assess the intra- and inter-rater reliability. However, due to low variability, kappa would be undeterminable for most of the comparisons; therefore, such an analysis was not performed.

One strength of our study is that three different experienced CT specialists assessed each puncture, performed twice and in a blinded fashion. Further, the use of the blood puddings is advantageous as it resembles the firmness of solid tumor lesions. The main weakness of our study is that the method is not evaluated in vivo; therefore, it is not known whether a contrast media trace can be seen and for how long in a perfused target lesion. On the other hand, any possible transient trace of biopsy (detected by repeated imaging) could indicate that the sample was taken from a desired perfused (non-necrotic) area. In addition, even though not observed in this in vitro study and probably of minor importance due to a short time span between biopsy and post-biopsy imaging, there may be a risk of sampling location overestimation in vivo due to the diffusion of contrast media to the interstitial space of the surrounding tissues. Further, it is not known how the method would perform in the border zone between solid tumors and more loose tissue, for example, normal lung or in necrotic tumors.

The major advantage with the method is the potential to reduce the number of repeat biopsies, given the associated complication risks and additional costs. These possible benefits, on the other hand, must outweigh the added cost for each biopsy procedure, in terms of the additional disposable items and the extra time spent on priming the biopsy needles. When performing transthoracic needle biopsies, the repeated priming procedure (when more than one biopsy is performed) would also mean an increased time of coaxial needle across the pleura, which is a known risk factor for pneumothorax (9). The administration of contrast media through the biopsy needle would also add a small, but not completely negligible, risk of allergic reactions. Further, an unlikely, but not entirely impossible, interference in the histopathology tissue processing related to the addition of highly concentrated iodine contrast media has been identified.

In conclusion, highly attenuating traces, sufficient to determine the sample locations after CT-guided biopsy, have been visualized in vitro by using our novel method of priming CBNs with contrast media, where using highly concentrated contrast media has been advantageous. The results first need to be confirmed in vivo before the clinical value of the method can be assessed.
